# A panel sequencing dataset of peripheral blood gene variations in pan-cancer

**DOI:** 10.1038/s41597-024-03620-6

**Published:** 2024-07-20

**Authors:** Yanxia Liu, Jie Liu, Shouwei Zhang, Jinyue Wang, Zhihong Sun, Huaibo Sun, Ying Yang, Guangmin Zheng, Yu Huang, Meng Li, Zhaojun Zhang, Jingfa Xiao, Changqing Zeng, Chengming Sun, Hongzhu Qu, Xiangdong Fang

**Affiliations:** 1 China National Center for Bioinformation, Beijing, 100101 China; 2 Beijing Institute of Genomics, Chinese Academy of Sciences, Beijing, 100101 China; 3 Yantai Yuhuangding Hospital, Yantai, 264000 China; 4 Genecast Biotechnology Co., Ltd, Wuxi, 214104 China; 5 National Genomics Data Center, China National Center for Bioinformation, Beijing, 100101 China; 6 University of Chinese Academy of Sciences, Beijing, 100049 China; 7 Beijing Key Laboratory of Genome and Precision Medicine Technologies, Beijing, 100101 China

**Keywords:** Genetic databases, Cancer genomics

## Abstract

Circulating cell-free DNA (cfDNA) in the peripheral blood is a promising biomarker for cancer diagnosis and prognosis. Somatic mutations identified in cancers have been used to detect therapeutic targets for clinical transformation and individualize drug selection, while germline variants can predict a patient’s risk of developing cancer and drug sensitivity. However, no platform has been developed to analyze, calculate, integrate, and friendly visualize these pan-cancer cfDNA mutations deeply. In this work, we performed panel sequencing encompassing 1,115 cancer-related genes across 16,659 cancer patients, spanning 27 cancer types. We detected 496 germline variants in leukocytes and 11,232 somatic mutations in the cfDNA of all patients. CPGV (Cancer Peripheral blood Gene Variations), a database constructed from this dataset, is the first pan-cancer cfDNA database that encompasses somatic mutations, germline variants, and further comparative analyses of mutations across different cancer types. It bears great promise to serve as a valuable resource for cancer research.

## Background & Summary

Large-scale sequencing projects have dramatically enhanced our molecular understanding of cancers to the point where using genomic analysis to improve treatment outcomes seems promising^1–4^. A pan-cancer atlas provides a panoramic view of the oncogenic processes that contribute to human cancer, featuring hundreds of predisposing germline variants identified and linked to functional consequences, thus providing guidelines for variant classification and germline genetic testing in cancer^5–8^. Actionable genetic alterations in signaling pathways provide opportunities for targeted and combination therapies^9–11^. In addition, pan-cancer analysis will enable effective therapies in one cancer type to be extended to other types with similar genomic profiles^12^. Therefore, pan-cancer mutation resources are valuable for cancer research and medical care.

Cell-free DNA (cfDNA) is a degraded DNA fragment which is released into the plasma; therefore, it can provide a panoramic view of the tumor genome, overcome intratumor heterogeneity issues, and can be used for cancer screening, diagnosis, and monitoring^13–16^. With the development of sequencing technology, the sensitivity and accuracy of cfDNA genotyping have greatly improved. Due to its characteristics of non-invasive sampling and easy-to-obtain, cfDNA has become a potential molecule that has attracted much attention for clinical transformation^17–20^. In recent years, an increasing number of studies related to cfDNA have been published, which have provided great help in the treatment of tumors^21–25^. Subsequently, a comprehensive database of cfDNA fragmentation was created^26^. However, no database or platform has been developed to integrate germline variants and somatic mutation data from pan-cancer peripheral blood.

To address this gap, we developed a comprehensive resource, named CPGV, to integrate gene variations from pan-cancer peripheral blood samples. CPGV would serve as an invaluable resource for cancer research.

## Methods

### Database construction

**Fig. 1:**
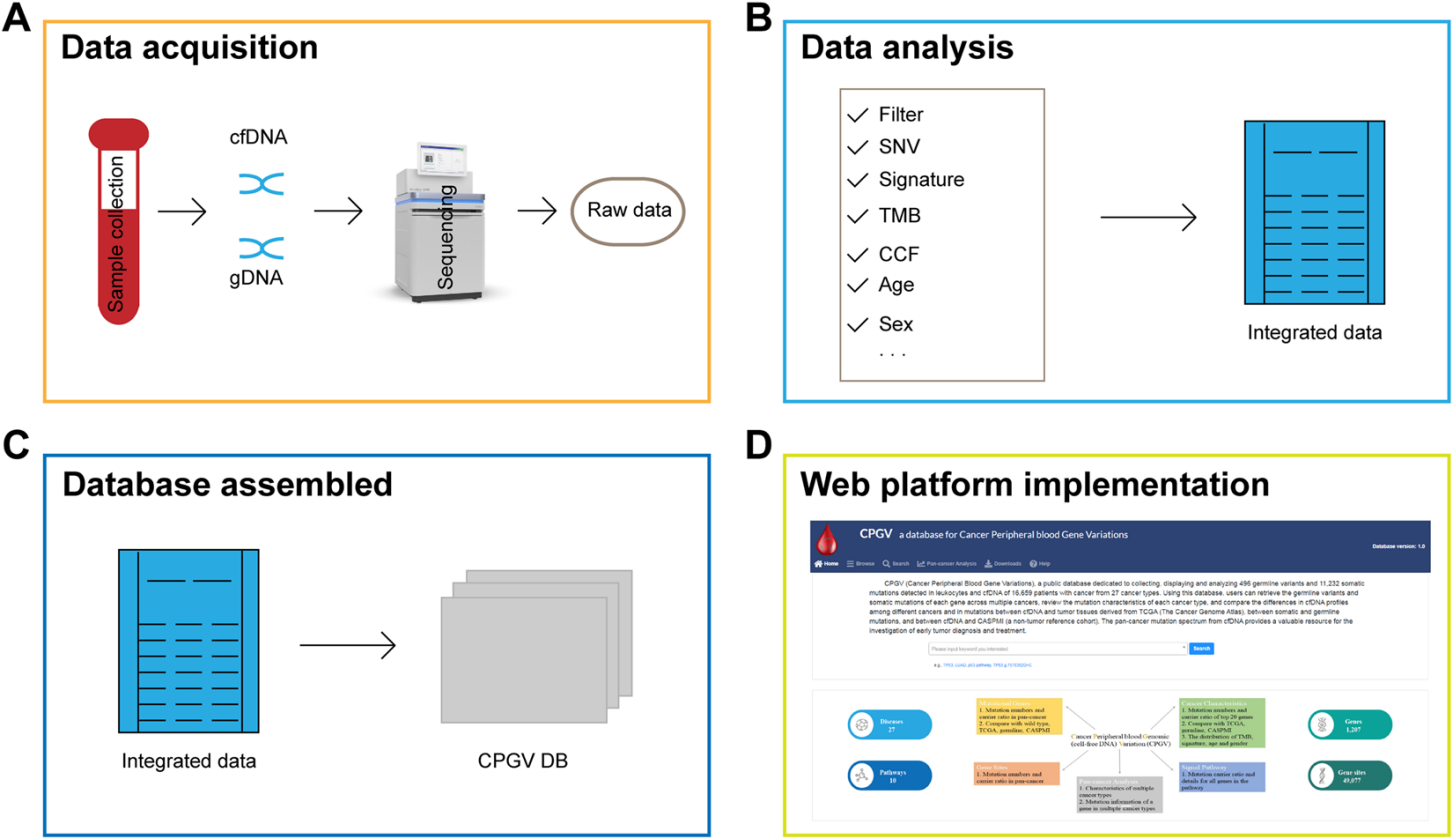
Overview of the CPGV DB construction method. (**A**) Data acquisition for pan-cancer peripheral blood gene variations. (**B**) Content of data analysis. (**C**) Database platform construction. (**D**) The CPGV DB web implementation.

First, we collected blood samples. Subsequently, cfDNA and genomic DNA (gDNA) were extracted and sequenced. Then, the raw data were uniformly filtered and analyzed. Finally, all data were assembled into the database system, and the web platform was implemented (Fig. 1).

### Sample collection

**Fig. 2:**
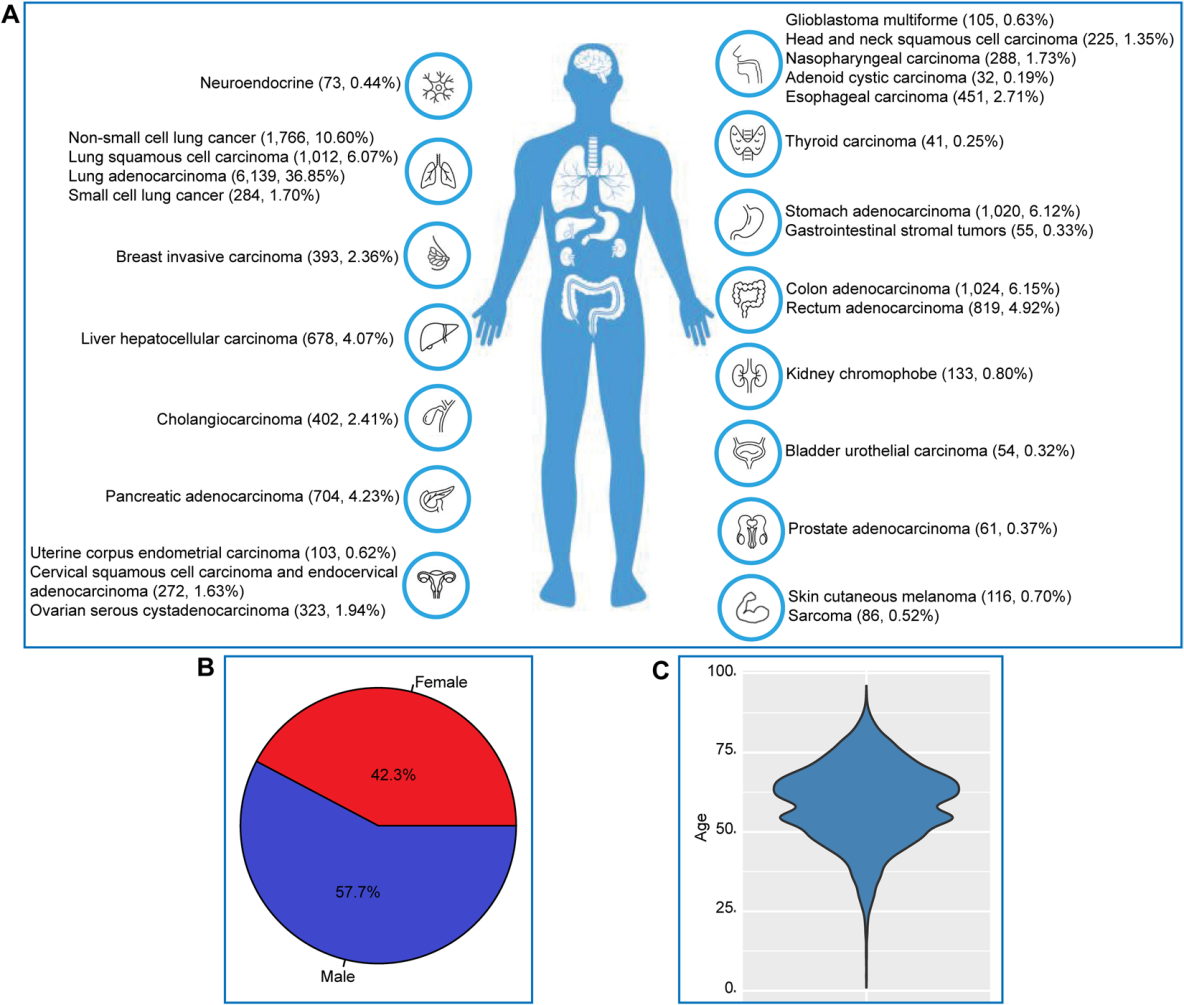
Statistics of the CPGV DB content. (**A**) The distribution of cancer types and numbers in the database. (**B**) The distribution of sex ratio. (**C**) The distribution of age.

Between September 2017 and March 2020, 16,659 patients with 27 cancer types were enrolled in this study (Fig. 2A). The ratio of males to females was approximately 3:2, with 42.3% (7,041) of the samples derived from females and 57.7% (9,618) from males (Fig. 2B). The age range was 1–96 years old, with an average age of 59 years old (Fig. 2C). Blood collections were performed twice for some patients at different time points (Supplementary Table S1), and we recruited 15,214 and 12,822 patients for somatic and germline research, respectively. All participants provided written informed consent before blood sampling, and guardians provided informed consent for any participants under 18 years of age. This study was reviewed and approved by the Ethics Committee of the Beijing Institute of Genomics, Chinese Academy of Sciences (Institutional Review Board No. 2021H016). The data has been filed with the Human Genetic Resource Administration of China and can be accessed under the data access agreement.

### DNA extraction, sequencing, and data processing

Plasma and white blood cells (WBCs) were separated by centrifugation at 1,600 × *g* for 10 min. cfDNA was extracted from plasma using a MagMAX™ Cell-Free DNA Isolation Kit (Thermo Fisher Scientific, Waltham, MA, USA). gDNA was extracted from WBCs using a TIANamp Blood DNA Kit (TIANGEN, Beijing, China). The DNA quality was assessed using an Agilent 2100 Bioanalyzer (Agilent, USA)^27^. gDNA was cut into 150–200 base-pair (bp) fragments with a Covaris M220 Focused-ultrasonicator (Covaris, Massachusetts, USA), constructed using the KAPA Hyper Prep Kit (Kapa Biosystems, USA)^28^, hybridized to several in-house panels (Genecast, Wuxi, China), and sequenced on the Illumina NovaSeq 6000 according to the manufacturer’s instructions, producing paired-end reads with a length of 151 bp.

Preliminary sequencing data in the BCL format were converted to FASTQ files using bcl2fastq (v2.20.0), processed using Trimmomatic (v0.39) for adapter trimming and low-quality read filtering^29^, mapped to the reference genome (hg19) using BWA (0.7.17)^30^, sorted and marked duplicates using the Picard toolkit (version 2.1.0)^31^, and then realigned using the Genome Analysis Toolkit (GATK, version 3.7)^32^.

### Somatic single nucleotide variation (SNV), germline single nucleotide polymorphism (SNP) and insertion-deletion (InDel) calling

GATK base quality score recalibration (BQSR) was first used to recalibrate base quality. For somatic SNVs and InDel calling, a panel of normals (PoN) containing germline and artifactual sites was created using GATK Mutect2 (4.1.2.0), and then Mutect2 was run to call variants in pairs of tumor and matched normal samples with the PoN. For germline SNP and InDel calling, GATK Haplotype Caller was used to call variants in normal samples in a joint calling mode. These variants were annotated with ANNOVAR and filtered using gnomAD for rare variants^33^. Rare variants were further filtered in the blacklist and healthy people, while nonsynonymous, stop-gain, stop-loss, splicing, frameshift/non-frameshift insertion, and deletion variants among the exonic and splicing regions were retained for later analysis. Pathogenicity classification for germline variants was predicted using CharGer following the American College of Medical Genetics guidelines^34^. Variants with a variant allele frequency (VAF) greater than 0.007 were retained for the final somatic mutation set.

### Mutation signature analysis

Mutation signatures were determined by applying somatic rare variants in parsing 96 tri-nucleotide contexts to calculate the proportion of the Catalogue Of Somatic Mutations In Cancer (COSMIC) signatures using the R package (version 4.1.2) “deconstructSigs”^35^.

### Calculation of tumor mutation burden (TMB)

TMB was determined by somatic mutations in the exonic and splicing regions with a VAF greater than 0.007. Alterations that were likely or known to be oncogenic drivers were excluded. TMB per megabase was calculated as the total number of mutations divided by the total bases of the target panel, with no less than 500x coverage.

### Estimation of circulating tumor DNA (ctDNA) content fraction (CCF)

The CCF of plasma samples was estimated using a maximum likelihood model based on SNVs and copy number variants in the paired plasma and WBC samples, a method that can calculate CCF at lower ctDNA concentrations with high accuracy and stability^36^.

### Calculation of gene-level variant number and carrier ratio for somatic and germline variants

Only the pathogenic and likely pathogenic germline variants predicted by CharGer were used to calculate the gene-level variant number and carrier ratio, and the dysfunctional variants among the exonic and splicing regions for somatic mutations were calculated. We counted the number of variants for each gene in the 27 cancer types. The carrier ratio of a variant is the percentage of patients with this variant per cancer type. The carrier ratio of a gene in a cancer is the proportion of individuals with any mutation in this gene among all patients with the same cancer type.

### Statistical analysis

The relationship between carrier ratio and cancer type in our cohort was determined by a one-sided Fisher’s exact test, and a two-sided Fisher’s exact test compared these relationships in our cohort and TCGA cohort and the somatic and germline in our cohort. Multiple hypothesis testing was carried out to adjust the p value.

## Data Records

VCF files recording all raw mutational data and the tumor classification of samples in this paper have been deposited in the Genome Variation Map^37^ (GVM) in the National Genomics Data Center^38^, China National Center for Bioinformation/Beijing Institute of Genomics, Chinese Academy of Sciences, under accession numbers GVM000186-GVM000195, GVM000197-GVM000207, GVM000209-GVM000227, and GVM000229-GVM000263 within the overarching project number PRJCA027469^39^ (https://ngdc.cncb.ac.cn/bioproject/browse/PRJCA027469). The raw sequence data reported in this paper have been deposited in the Genome Sequence Archive^40^ (GSA) in the National Genomics Data Center, China National Center for Bioinformation/Beijing Institute of Genomics, Chinese Academy of Sciences (GSA-Human: HRA004543^41^) that are publicly accessible at https://ngdc.cncb.ac.cn/gsa-human/browse/HRA004543. At the same time, these data can also be browsed, searched and compared through our online database website.

## Technical Validation

All samples adopt a unified data quality control and analysis process to ensure that different samples can be compared.

The data undergoes a series of rigorous filtering process, ensuring that only high-quality variants that meet all filtering criteria are included in the database. Low quality reads that meet the following conditions would be excluded: 1) Fisher Strand (FS) value exceeding 200.0; 2) Symmetric Odds Ratio (SOR) value greater than 3.0; 3) Mapping Quality (MQ) below 40.0; 4) MQRankSum value less than −12.5; 5) Quality by Depth (QD) value below 2.0; 6) QUAL value less than 30.0; 7) ReadPosRankSum value less than −20.0.

**Fig. 3:**
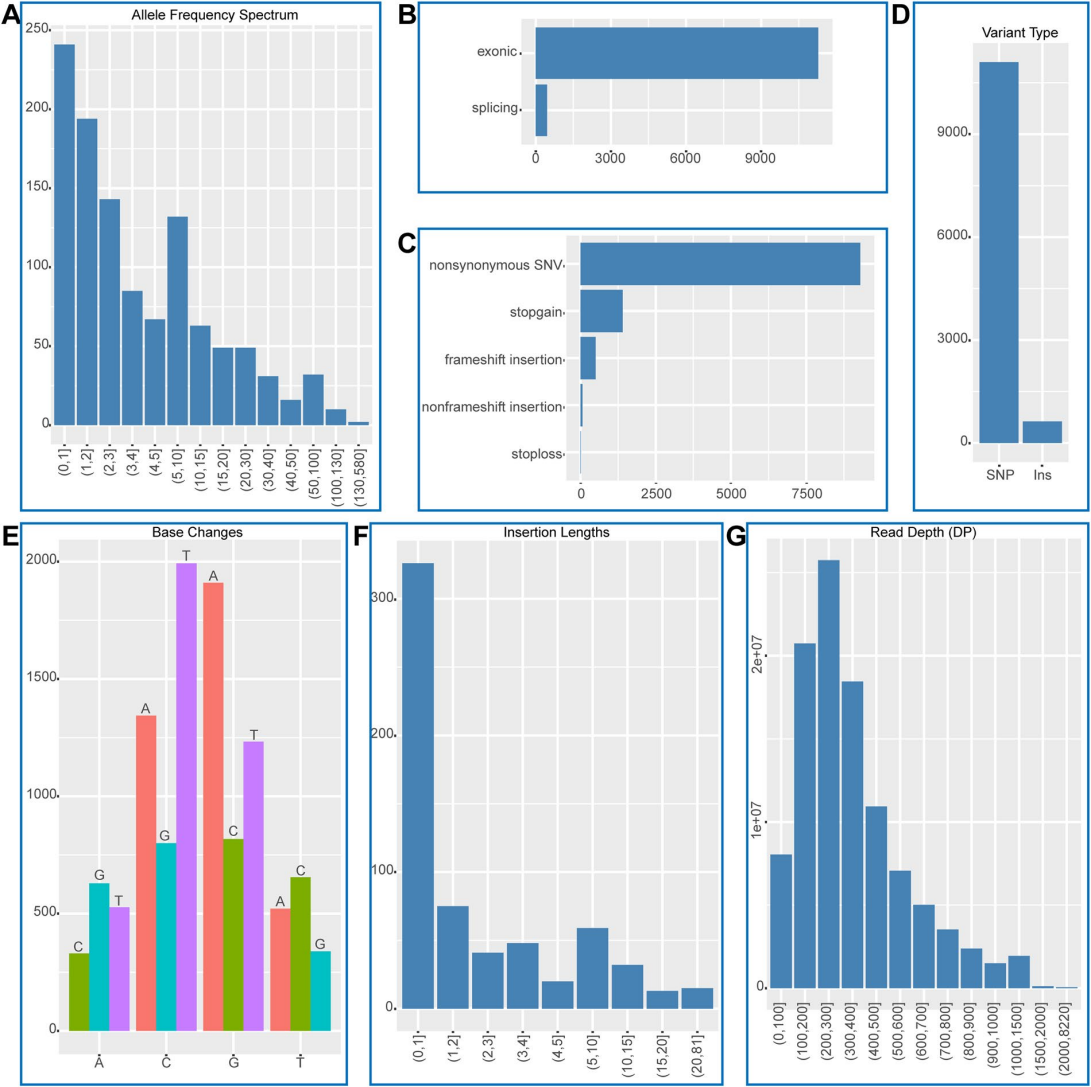
Summary of all mutation sites. (**A**) The distribution of the alternative allele frequency. (**B**) Annotation of genomic location. (**C**) Annotation of variant consequence. (**D**) Annotation of variant types. (**E**) The distribution of Nucleotide substitutions per base. (**F**) The distribution of insertion lengths. (**G**) The distribution of read depth.

Genes harboring only 1 mutation site account for 21.6%, while those with 5 or fewer mutations make up 65.5% (Fig. 3A). Approximately 96.2% of the mutations occur within the exonic region (Fig. 3B), 94.6% involve SNP variations, and nonsynonymous SNV constitute 82.4% (Fig. 3C,D). Specific mutation patterns reveal a tendency for A to G (42.3%), C to T (48.2%), G to A (48.2%), and A to C (43.2%) (Fig. 3E). Insertions are predominantly single-base event, comprising 51.8% of all insertions (Fig. 3F). The majority of reads, accounting for 71.9%, have depths ranging from 100 to 500 (Fig. 3G). For a more detailed overview, the statistics of somatic mutants and germline mutants are shown in Supplementary Figure S1, S2, respectively.

The purpose of constructing this database is to integrate genetic variation data derived from peripheral blood samples of cancer patients, providing a wealth of mutated gene sites for research. Notably, only 18% of our SNVs overlap with those listed in COSMIC^42^, the foremost and most comprehensive resource of somatic mutations in human cancer, suggesting the significant value of our dataset. In addition, compared with other databases, such as VARAdb^43^ and OncoVar^44^, our database offers distinct advantages. These include the provision of germline mutation site information, the capability to compare across multiple cancer types, and the facility to contrast our data with other datasets (Table 1). By leveraging these unique features, researchers can gain a more comprehensive understanding of genetic variations in cancer and identify potential therapeutic targets.

**Table 1:** Comparison of CPGV with other databases that provide searches for cancer gene variations.

Type	Function type	CPGV	VARAdb	OncoVar	COSMIC
Variations		11,728	577,283,813	20,162	23,854,105
Theme		peripheral blood gene variations in cancers	human variation annotation	oncogenic driver variants in cancers	catalogue of somatic mutations in cancer
Content	Annotation	√	√	√	√
	Mutations number/frequency	√	√	√	√
	Including germline mutations	√	√		
	Comparison with wild type	√	√		√
	Comparison with other datasets	√			
	Comparison across multiple cancer types	√			√
	Clinical information	√	√	√	√

## Usage Notes

Both the processed mutation data stored in GVM and the raw data stored in GSA-Human are open to users for free. However, due to the sensitivity of human genetic data, users need to apply on the GVM platform or GSA-Human platform and fill in the data access agreement before downloading the data. The data at GVM is held under the same terms and conditions as the data at GSA-Human. The user’s application will be approved provided that the user agrees to the terms and conditions of the data access agreement and the purpose is not for commercial profit. In addition to the repository data, our dataset is also available for users to download and visualize at http://ngdc.cncb.ac.cn/cpgv/.

Users need to register an account first, log in to find the data they are willing to request by entering the access number in the search bar, then click the “Request” button, and follow the steps to make their Data Access Request. The data access agreement can be downloaded during the intermediate process. Additionally, users can refer to the “Guidance for Making Data Access Requests” under the document button in the navigation bar for further assistance on submitting their requests. Once the Data Access Committee (DAC) has approved the request, users will receive an email notification from GVM/GSA confirming the approval. Upon receiving this notification, users can log in to their account, click on “Request” in the navigation bar, and select “My requests” to check the status of their application. By clicking on “view” in GVM or “download” in GSA, they will be able to access the data download address.

### Supplementary information

Supplementary information 1

Supplementary information 2

## Data Availability

The code of the CPGV has been uploaded to GitHub: https://github.com/padapeng911/CPGV.
