# Supplementary material for: A panel sequencing dataset of peripheral blood gene variations in pan-cancer
Source: Sci Data. 2024 Jul 20;11:805. doi: 10.1038/s41597-024-03620-6 (PMC11271301; doi:10.1038/s41597-024-03620-6)
Supplement: Supplementary file 1 — Supplementary information 1 [file 41597_2024_3620_MOESM1_ESM.pdf]

Supplementary Figure S1 Summary of somatic mutation sites………………………………......2
Supplementary Figure S2 Summary of germline mutation sites…………………………………3

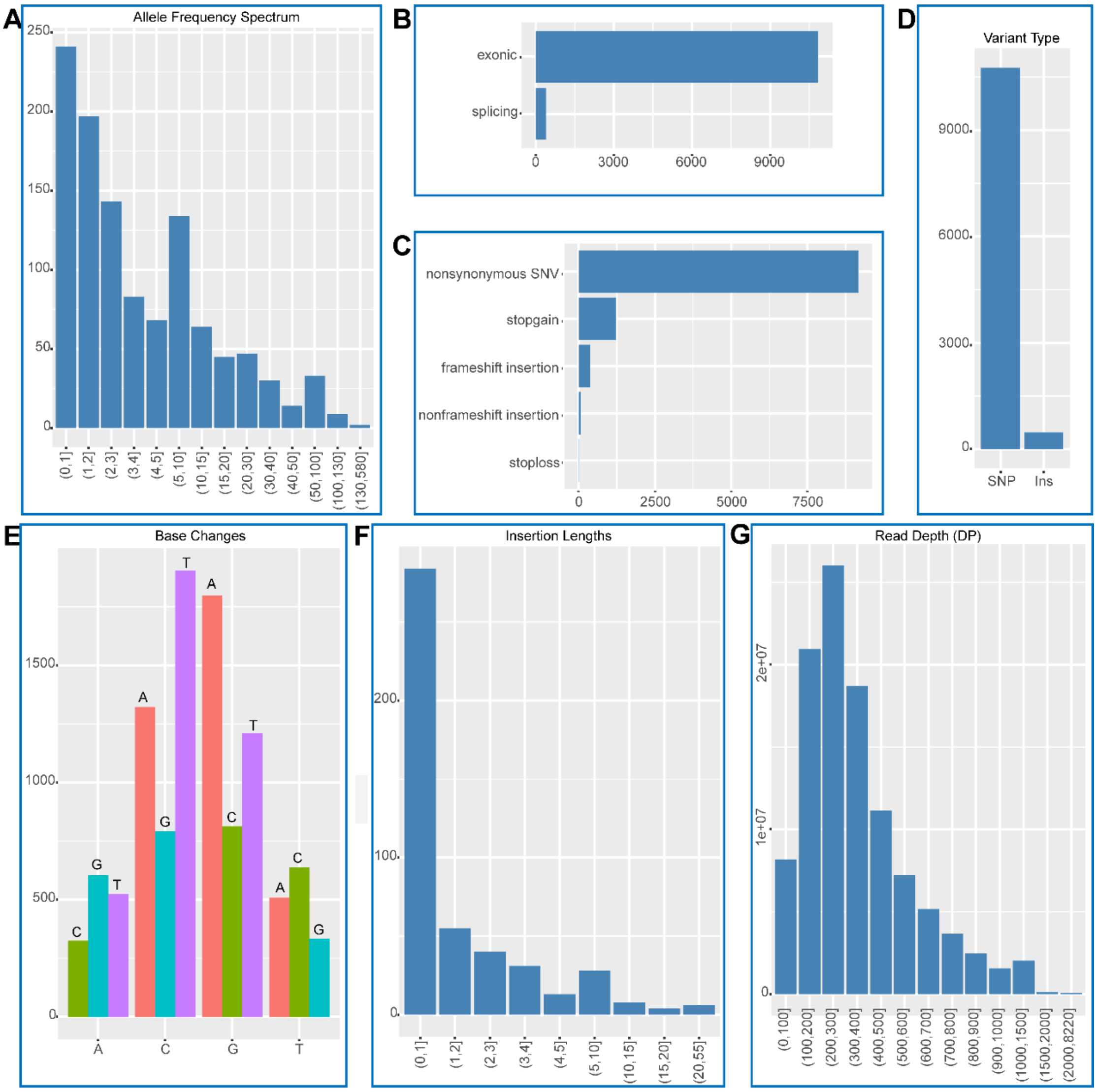

Supplementary Figure S1 Summary of somatic mutation sites. (**A**) The distribution of the alternative allele frequency. (**B**) Annotation of genomic location. (**C**) Annotation of variant consequence. (**D**) Annotation of variant types. (**E**) The distribution of Nucleotide substitutions per base. (**F**) The distribution of insertion lengths. (**G**) The distribution of read depth.

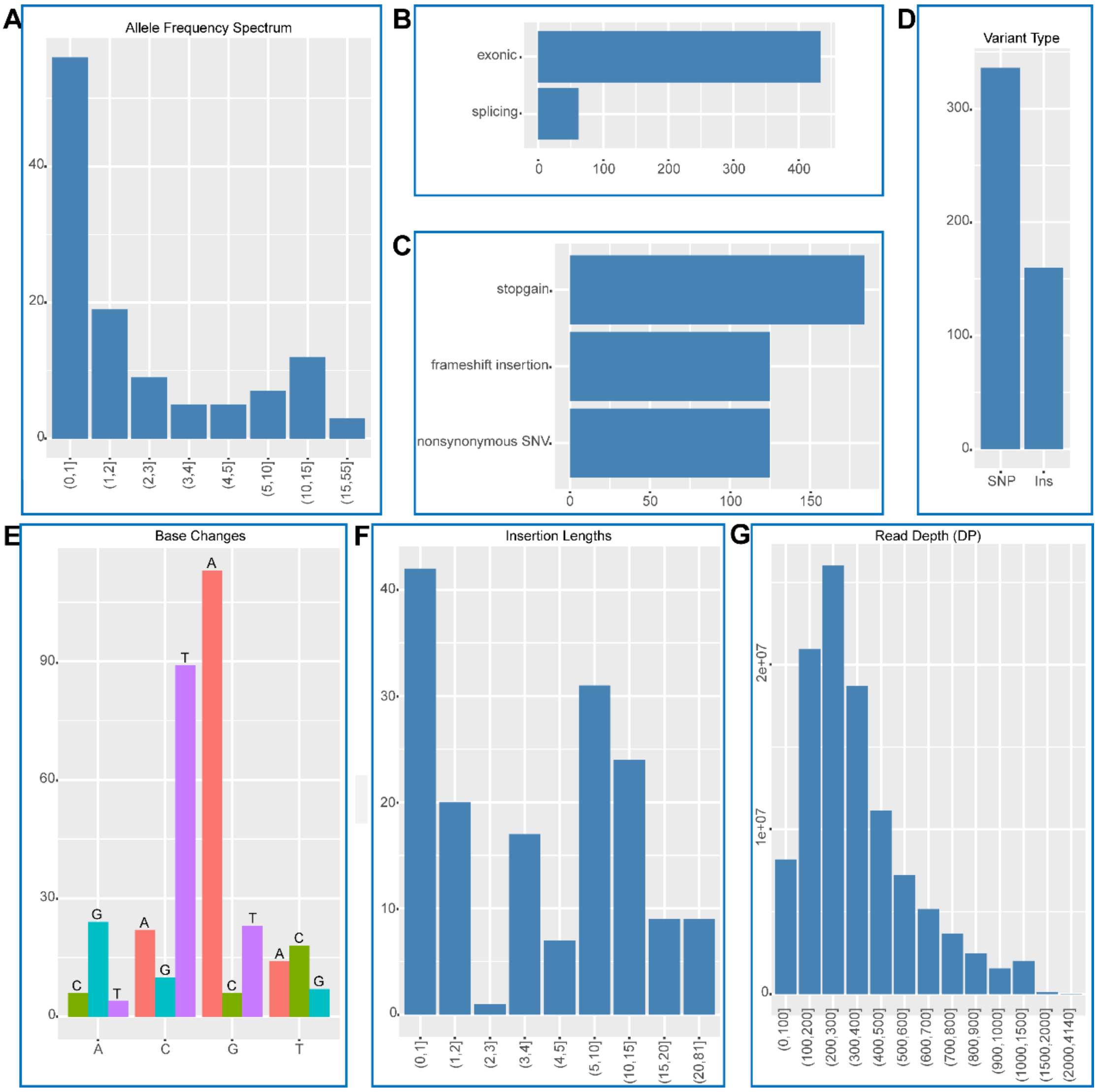

Supplementary Figure S2 Summary of germline mutation sites. (**A**) The distribution of the alternative allele frequency. (**B**) Annotation of genomic location. (**C**) Annotation of variant consequence. (**D**) Annotation of variant types. (**E**) The distribution of Nucleotide substitutions per base. (**F**) The distribution of insertion lengths. (**G**) The distribution of read depth.
